# Instances of the Medicinal Effects of Magnetism

**Published:** 1782

**Authors:** 


II. *Inſtances of the medicinal effects of Magnetiſm. Communicated in a letter to Dr.* Simmons, F. R. S. *by Mr.* Thomas Henry, F. R. S. *Apothecary at Mancheſter. Read* Sept. 9, 1782.

IN the laſt number of the London Medical Journal ſome account is given of the medicinal effects of magnetiſm. Perhaps the following caſes, which have fallen within my own obſerobfervation, may be of fome ufe in recommending a trial of it. That it is a powerful agent in nature, we all know, though we are little acquainted with the modus operandi.

A young gentleman had been for fome days troubled with a very fevere tooth-ach, for which he had tried all the ufual remedies without fuccefs, and was on the point of fubmitting to the extraction of the tooth; when a friend informing him that the application of a magnet had been known to effect a cure, he immediately purchafed a fmall artificial one, fuch as thofe fold in the fhops for tobacco-ftoppers, and with little expectation of fuccefs, applied it to his tooth. To his great furprize, in a few minutes, the pain entirely ceafed, nor had he any return of it afterwards.

Being myfelf afflicted, laft winter, with fevere pain in a decayed tooth, which was too rotten to be eafily extracted, and having tried various remedies in vain, I recollected the above cafe, and, having a magnet in the houfe, applied it to the tooth. Inftant relief fucceeded, my pain left me; and though it afterwards returned feveral times in the courfe of a few days, it was conftantly removed by the magnet, which I carried for that purpofe in my pocket, and I have fince remained quite free from it.

About ſix weeks ſince a tinnitus aurium was very troubleſome to me every night. At firſt I only perceived it when I lay on my right ſide, on which was the affected ear; but it ſoon increaſed ſo as to diſturb me on whichever ſide I reclined. One night, being much diſturbed with it, and having nothing near that ſeemed likely to relieve me, except the magnet, I determined to apply it, and introducing it into my ear, and holding it there for ſome minutes, when I again lay down I was free from the noiſe, nor did it return for ſeveral nights. A ſlight attack which happened, in the courſe of that week, was inſtantly removed by the ſame means, ſince which time I have been perfectly relieved from my complaint.

